# Control strategies to re-establish glenohumeral stability after shoulder injury

**DOI:** 10.1186/2052-1847-5-26

**Published:** 2013-12-06

**Authors:** Bala S Rajaratnam, James CH Goh, Prem V Kumar

**Affiliations:** 1 School of Health Sciences (Allied Health), Nanyang Polytechnic, 180 Ang Mo Kio Avenue 8, 569830, Singapore; 2 Faculty of Medicine, National University of Singapore, Singapore

**Keywords:** Electromyography, Anterior shoulder instability, Elevation, Neuro-motor control

## Abstract

**Background:**

Muscles are important “sensors of the joint instability”. The aim of this study was to identify the neuro-motor control strategies adopted by patients with anterior shoulder instability during overhead shoulder elevation in two planes.

**Methods:**

The onset, time of peak activation, and peak magnitude of seven shoulder muscles (posterior deltoid, bilateral upper trapezius, biceps brachii, infraspinatus, supraspinatus and teres major) were identified using electromyography as 19 pre-operative patients with anterior shoulder instability (mean 27.95 years, SD = 7.796) and 25 age-matched asymptomatic control subjects (mean 23.07 years, SD = 2.952) elevated their arm above 90 degrees in the sagittal and coronal planes.

**Results:**

Temporal characteristics of time of muscle onsets were significantly different between groups expect for teres major in the coronal plane (t = 1.1220, p = 0.2646) Patients recruited the rotator cuff muscles earlier and delayed the onset of ipsilateral upper trapezius compared with subjects (p<0.001) that control subjects. Furthermore, significant alliances existed between the onsets of infraspinatus and supraspinatus (sagittal: r = 0.720; coronal: r = 0.756 at p<0.001) and ipsilateral upper trapezius and infraspinatus (sagittal: r = -0.760, coronal: r = -0.818 at p<0.001). The peak activation of all seven muscles occurred in the mid-range of elevation among patients with anterior shoulder instability whereas subjects spread peak activation of all 7 muscles throughout range. Peak magnitude of patients’ infraspinatus muscle was six times higher (sagittal: t = -8.6428, coronal: t = -54.1578 at p<0.001) but magnitude of their supraspinatus was lower (sagittal: t = 36.2507, coronal: t = 35.9350 at p<0.001) that subjects.

**Conclusions:**

Patients with anterior shoulder instability adopted a “stability before mobility” neuro-motor control strategy to initiate elevation and a “stability at all cost” strategy to ensure concavity compression in the mid-to-150 degrees of elevation in both sagittal and coronal planes.

## Background

Despite having undergone a shoulder stabilization procedure, 31% or 5 out of 16 patients experienced post-operative dislocation [1]. Less invasive procedures such as arthroscopic stabilization of the shoulder decreased the recurrence of dislocation to less than 22% [2]. Thus, post-operative dislocation is an issue and one potential cause is persistent muscle imbalance and altered muscle activation after surgical correction [3-8].

When the passive structures of the shoulder such as capsule and labrum are damaged, the central nervous system signals the rotator cuff muscles to re-establish sufficient concavity compression for glenohumeral stability. For instance, when the supraspinatus is torn, the changes in magnitude and abnormal activation patterns of the other shoulder muscles centered the head of the humerus within the glenoid fossa and resisted the upward pull of the delotids [9,10]. Thus, quantifying the neuro-motor control strategies at the unstable shoulder could lead to better rehabilitation management of the injured shoulder after surgery.

Patients with anterior shoulder instability (ASI) had less activities of the pectoralis major, biceps brachii, supraspinatus and subscapularis, more peak activity of infraspinatus and slower biceps brachii reflex latency during elevation [6]. Glousman and colleagues found that during a throwing activity, subjects with glenohumeral instability increased the force magnitude of their biceps and supraspinatus and recruited less pectoralis major, subscapularis, latissimus dorsi and serratus anterior [11]. However, most recurrences of shoulder dislocation occur primarily in the overhead apprehension position. Based on estimates of muscle force magnitudes from electromyography studies, the lines of action of resultant force vectors in the apprehension position are more anterior and at their lowest level for glenohumeral stability, explaining why a quick and trivial action involving minimal force such as putting on a T-shirt may lead to repeated dislocations after ASI [12,13]. These findings seem to suggest the need to selectively strengthen the muscles of glenohumeral joint to re-establish shoulder stability.

Findings from studies that quantified muscle activation patterns among patients with spinal dysfunction led to changes in rehabilitation programs and better functional outcomes [14,15]. The current rehabilitation strategy to manage the unstable shoulder is to strengthening the rotator cuff and scapula-thoracic muscles. Rehabilitation without correcting inappropriate neuro-motor muscle patterns or cortical maps after surgical correction of instability could facilitates post-operative dislocation [16,17]. Thus, quantifying the recruitment characteristics of shoulder muscles during unconstrained arm elevation in persons with ASI allows us to understand the feed forward and feedback controls strategies that regulate glenohumeral stability throughout the range of arm elevation. Such information is lacking and could lead to more effective rehabilitation programs to better manage the unstable shoulder and minimizes post-operative dislocation [18].

The aim of this biomechanical study was to quantify temporal and magnitude characteristics of shoulder muscle activation patterns of patients with ASI as they performed everyday overhead tasks in two planes of arm elevation. Their results were compared with asymptomatic subjects to determine differences in neuro-motor control strategies to perform these everyday tasks.

## Methods

The Institutional Review Board of the University approved this study (DSRB-D/00/863).

### Patients and subjects

This study recruited 19 pre-operative patients (mean age: 27.95; SD = 7.796; 16 males and 3 females) who experienced traumatic ASI more than 6 months earlier. It has been reported that patients whom have experienced temporary lesions to the axillary, suprascapular and musculo-cutaneous nerves would have regain full nerve conduction within 6 months after injury [19].

The Orthopedic Surgeon involved in this study confirmed that all patients had a Type 1 injury (True TUBS -Traumatic Unilateral Bankart lesion treated with surgery) based on the Stanmore classification [5]. All patients had at least three episodes of recurrent dislocations/subluxations after the initial trauma. The exclusion criteria included those who had prior shoulder surgery and fractured their affected upper limb, and experienced shoulder pain. Those who demonstrated unresolved nerve damages by demonstrating greater that 10% difference in bilateral grip strength between both hands were also excluded from this study [20].

Control subjects were 25 young age-match male individuals (mean age 23.07; SD = 2.952) who had no history of shoulder pathology, upper limb neuromuscular dysfunction, neurological deficits or cardiac disorders. Both patients with ASI and control subjects signed informed consents before participating in the study.

### Assessment of patients with ASI

The author examined all patients with ASI and subjects before data collection. He palpated their acromioclavicular and glenohumeral joints, rotator cuff tendons and biceps tendons for tenderness or localized pain. Thereafter, he tested the laxity of their thumbs, elbows and knees and found no signs of hyperlaxity based on the Beighton Hypermobility Score (score of 0 at all three joints bilaterally). Next, he quantified their active range of shoulder rotation with a goniometer, and the strength of their shoulder rotators and deltoid muscles in position suggested by the Kendall’s manual muscle testing procedures. He also performed the Apprehension test, Neer test, Sulcus sign test, Speed test and Empty Can test to identify underlying shoulder pathologies of patients (Table 1).

**Table 1:** Physical assessment of patients with Anterior Shoulder Instability

	**Anterior shoulder instability (n = 19)**
ROM in degrees at 90 degree abduction	
• External rotation (SD : Range)	74.2 (6.07: 62–83)
• Internal rotation (SD : Range)	62.2 (12.08: 41–70)
Muscle strength measured in MMT# grades (SD) of:	
• External rotators	4.8 (0.36)
• Internal rotators	4.2 (0.41)
• Deltoids	4.7 (0.46)
Special test (numbers of patients):	
• Apprehension	+ ve (15) - ve (4)
• Neer	+ ve (6) - ve (13)
• Sulcus grade	Gd 3 (14) Gd <3 (5)
• Speeds	+ ve (2) - ve (17)
• Empty can	+ ve (9) - ve (10)
*WOSI score (SD)/2100	614.7 (277)

*WOSI: *The Western Ontario Shoulder Instability Index.*

# *MMT*: *Kendall*’*s Manual Muscle Testing procedure*.

The Apprehension test was conducted with patients lying in supine with their arms in external rotated, abduction and slight extension. Presences of pain and/or apprehension during this test (positive) suggest the likelihood of ASI. The Neer test was performed in the sitting position as the author limited the patient’s scapular rotation while their affected arm was passively rotated internally during elevation in the scapular plane. A positive Neer test indicates likely presence of subacromial impingement. Patients were seated with their arms at the side to perform the Sulcus sign test. A distracting force was applied to their arm to grade displacement of the acromion from the greater tuberosity. Sulcus sign test grade of 3 (>2.0 cm) suggest multidirectional glenohumeral instability. The Speed test was done in sitting with the patient’s elbow in extension, forearm supinated and the humerus elevated to 60 degrees while the tester resisted humeral forward flexion. A positive test indicates deficits to the long head of the biceps or biceps/labral complex. The Empty can test was also performed in sitting with the humerus at 90 degrees of forward flexion in the plane of the scapula (approximately 30 degrees of abduction), full shoulder internal rotation with the thumb pointing down while the patient resisted downward pressure applied by the tester to the superior aspect of their distal forearm. Pain and weakness during the test indicates a torn supraspinatus muscle. Finally, all patients completed the Western Ontario Shoulder Instability questionnaire (WOSI), which consisted of 21 questions related to their physical symptoms, and their quality of life and emotions during sports/recreation/work and lifestyle after experiencing recurrent shoulder instability [21].

### Electromyography (EMG) placement and detection

The skin over the shoulder of all patients and subjects was cleaned with alcohol swabs, and two bi-polar surface Ag/AgCl adhesive electrodes of size less 50 mm^2^ were placed no more than 20 mm apart from center to centre to collect sEMG signal outputs of the posterior deltoid, bilateral upper trapezius and biceps brachii. Intramuscular fine-wires were prepared using the method described by Park & Harris [22] and Morris and colleagues [23]. Two 25 μm-diameter Teflon-coated wires [a] were inserted into a single 25-gauge hypodermic needle before sterilization. Intramuscular fine-wires were inserted into the muscle belly of infraspinatus, supraspinatus and teres major. The location of both surface and fine-wire electrodes placement were recommendations by Cram and Kasmen [24] and Perotto [25] respectively to minimal cross talks.

Motion artifact and signal noises were minimized by securing and anchoring cables and electrodes. The reference earth electrode was placed on a bony landmark away from the experimental shoulder [26]. For subjects, their experimental shoulder was their dominant hand. All subjects and patients were positioned in the optimal muscle testing position recommended by Hislop and Montgomery [27] and 10 seconds of maximal voluntary isometric contraction (MVC) recorded for each muscle being studied. Each muscle’s peak magnitude as normalized as a percentage of its MVC value.

Electrodes were connected to a Motion Lab MA316 [b] pre-amplified double-differential input connector (common-mode rejection ratio [C.M.R.R] 110 dB at 65 Hz and gain of 20% at 1 KHz). The double-differential input connectors had an impedance of greater than 100 meg ohms and a built-in noise filter of less than 1.2 μV. EMG signals were collected by a Windaq DI-710 stand-alone data logger [c]. The bandwidths of sEMG and fEMG signals were the same and thus, both signals were filtered at 10–100 Hz to allow concurrent comparison between electrode types. The sampling rate of EMG signals for all muscles was 1800 samples per second. Signals were amplified with a gain of 10. All signals were stored in a computer for off-line analyses.

The Shewhart single threshold criterion method was chosen to identify muscle onset. Staude [28] found this method could identify signals within a 100 ms window with 99.9% accuracy and a mean error of −7.1 ms for time sensitive signals. The single muscle onset threshold of one standard deviation above the mean baseline magnitude lasting greater than 25 ms criterion had a strong likelihood of committing a Type I error [29,30] while 3 standard deviations cut-off resulted in a Type II error [29,31,32]. Thus, this study established the time of muscle onset as the period when the signal was two standard deviations above the mean baseline magnitude, lasting 25 ms and with signal-to-noise ratios of greater than four displayed on the Windaq Waveform Browser for MMC. The time when the muscle reached peak magnitude was also identified with a signal detection programme written with MATLAB [d] software. Next, time sensitive muscle onset and peak magnitude of each muscle were normalized between trails and subjects.

### Data collection procedure

**Figure 1:**
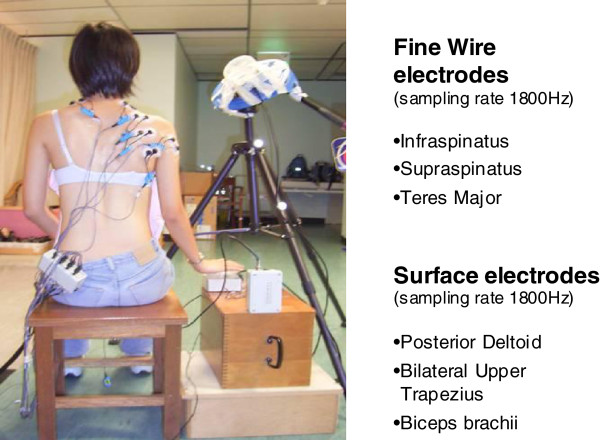
Experimental set-up.

Patients with ASI/control subjects sat on a chair without an armrest or backrest and with their feet flat on the ground. Their affected or dominant hand rested on a light switch pad positioned by their side of the arm. On instruction, they raised their hands at their normal speed to tap a second switch pad placed within reaching distance and in the coronal plane (abduction) at 150 degrees of elevation (Figure 1).

Next, they performed the same action to tap the second switch but now placed in the sagittal plane (flexion). Data of nine trials for each patient/subject in each plane was collected. Signals from the switches identified movement onset and termination and this permitted normalization of time between trials and subjects.

### Statistical analysis

SPSS Version 17.0 for Windows package [c] was used to evaluate the data. A *t*-test for independent variables differentiated the mean times of muscle onsets, times of peak muscle activations and peak muscle magnitudes between patients and subjects. Pearson correlations examined muscle alliances in the pre-setting phase (before movement start), in the setting phase (0 to 60 degrees), the mid-range (61 to 120 degrees) and the end-range (121 to 150 degrees) of elevation. Statistics significance was set at p < 0.05.

## Results

### Physical assessment

Table 1 summarizes the results of physical assessment of patients with ASI. The mean WOSI score indicate that patients cope well with their physical dysfunction even though 36% of them experience shoulder impingement during arm elevation.

### Time of muscle onsets

Table 2 highlights that temporal characteristics of times of muscle onsets were significantly different between groups expect for teres major in the coronal plane (t = 1.1220, p = 0.2646). Patients recruited the supraspinatus and infraspinatus early and this neuro-motor action delayed the activation of ipsilateral upper trapezius activity. Another noticeable feature was that patients with ASI activated their contralateral upper trapezius earlier in the sagittal plane of elevation.

**Table 2:** Onset times of muscle activations between Patients with ASI and Control young subjects during elevation in the sagittal and coronal planes

**Muscles**	**Sagittal plane**				**Coronal plane**			
	**Patients with ASI (time as a %)**	**Control young (time as a %)**	**t-test values**	**p-value**	**Patients with ASI (time as a %)**	**Control young (time as a %)**	**t-test values**	**p-value**
	**(SD)(SE)**	**(SD)(SE)**			**(SD)(SE)**	**(SD)(SE)**		
Teres major	41.03 (12.63) (1.31)	50.22 (5.13) (0.41)	6.64	<0.001*	47.64 (20.05) (2.09)	50.04 (5.27) (0.43)	1.12	
Supraspinatus	−21.98^#^ (8.90) (0.92)	22.31 (2.29) (0.18)	46.74	<0.001*	−22.60^#^ (6.86) (0.71)	21.04 (2.20) (0.17)	59.14	<0.001*
Infraspinatus	8.00 (1.62) (0.16)	60.29 (6.05) (0.49)	100.12	<0.001*	8.35 (1.64) (0.17)	56.86 (5.85) (0.47)	95.45	<0.001*
Posterior deltoid	35.36 (11.22) (1.17)	9.28 (0.95) (0.07)	−22.23	<0.001*	40.93 (13.25) (1.38)	9.34 (0.92) (0.07)	−22.83	<0.001*
Ipsilateral upper trapezius	9.61 (6.55) (0.68)	−2.60^#^ (0.26) (0.02)	−17.88	<0.001*	15.80 (16.33) (1.70)	−2.60^#^ (0.27) (0.02)	−10.81	<0.001*
Contralateral upper trapezius	32.36 (10.64) (1.11)	82.20 (7.38) (0.60)	39.45	<0.001*	41.63 (12.77) (1.33)	81.86 (8.56) (0.69)	25.84	<0.001*
Long head of biceps	56.22 (14.56) (1.51)	80.70 (8.18) (0.66)	17.23	<0.001*	49.43 (11.94) (1.24)	82.27 (9.33) (0.76)	29.51	<0.001*

*Significant at p < 0.001; # -ve values indicate muscle activation before movement start (i.e. pre-setting phase).

Among patients with ASI, the time of muscle onsets between ipsilateral upper trapezius and infraspinatus in both planes were negatively correlated (sagittal: r = −0.760, coronal: r = −0.818 at p < 0.001). Time of muscle onset between supraspinatus and infraspinatus in both planes were positively correlated (sagittal: r = 0.720, coronal: r = 0.756 at p < 0.001).

### Time and Peak muscle magnitude

**Figure 2:**
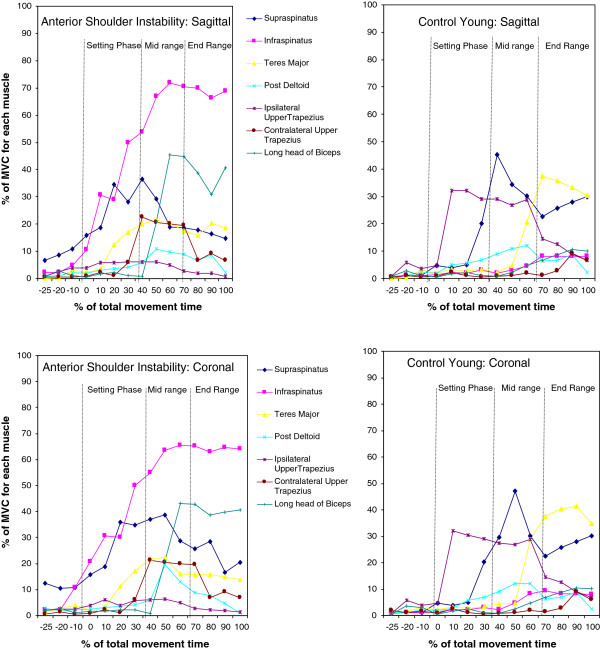
Trends of magnitudes of muscle activations between patients with anterior shoulder instability and control young subjects during elevation in the sagittal and coronal planes.

Times of peak magnitude of all muscles except for suprapsinatus between control subjects and patients with ASI were also statistically different. The time of supraspinatus activation during elevation in the sagittal plane between groups were similar (t = −1.93023, p = 0.0566). Patients with ASI recruited peak muscle activation of all seven muscles in the mid-range of elevation in both planes, while control subjects spread peak activations throughout the range of shoulder elevation (Figure 2).

**Figure 3:**
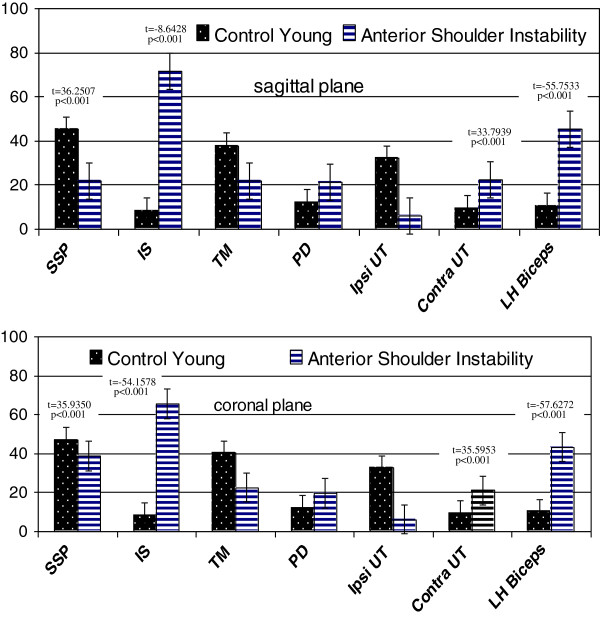
Peak magnitudes of muscles activation between anterior shoulder instability and control young subjects during elevation in the sagittal and coronal planes.

Peak magnitude of infraspinatus among patients with ASI was six times higher than control subjects in both planes (sagittal: t = −8.6428, coronal: t = −54.1578 at p < 0.001). Peak magnitudes of contralateral upper trapezius (sagittal: t = 33.7939, coronal: t = 35.5953 both at p < 0.001) and biceps were statistically higher in both planes (sagittal: t = −55.7533, coronal: t = −57.6272 both at p < 0.001) while supraspinatus was statistically lower among patients with ASI compared to control subjects (sagittal: t = 36.2507, coronal: t = 35.9350 both at p < 0.001) (Figure 3).

## Discussion

This biomechanical study identified different neuro-motor control strategies that the unstable shoulder adopted to maintain glenohumeral stability after injury. Patients with ASI demonstrated a “stability before mobility” strategy in the pre-setting phase and “stability at all cost strategy” in the mid-range of arm elevation to ensure successful elevate of their arm overhead in both planes of arm elevation. These strategies were different to those adopted by control subjects. It has been reported that the central nervous system prefers fixed strategies to elevate the arm overhead in all planes, probably to simplify neuro-motor control [33]. To the best of our knowledge, no study has quantified the neuro-motor control strategies of overhead arm motion in different planes of arm elevation among pre-operative patients with ASI.

### Stability before mobility

**Figure 4:**
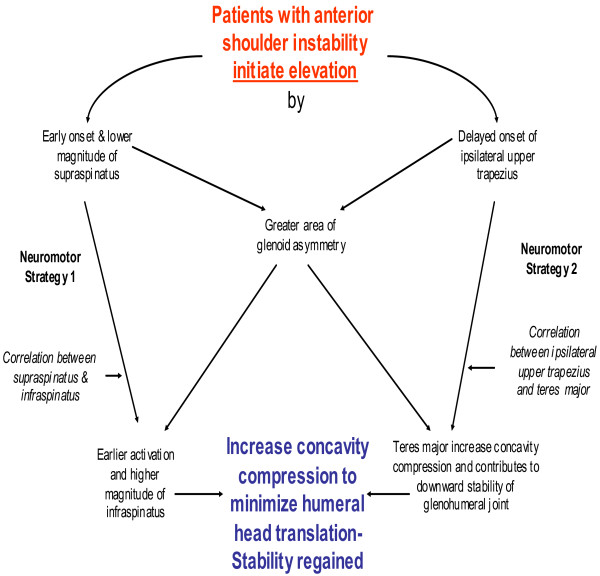
Hypothesis neuro-motor control strategies that patients with anterior shoulder instability adopt in setting phase of arm elevation.

Patients with ASI activated their supraspinatus first, as early as 22% before the onset of movement compared with control subjects (Figure 4). By selecting the “stability before mobility” neuro-motor control strategy, they quickened their reflexive response and improved the effectiveness of achieving successful glenohumeral stability before commencing arm elevation [6]. The early recruitment of supraspinatus pulled the humeral head posteriorly into a closed-pack position and counteracted the upward and anterior shear forces generated by the contracting anterior and middle deltoids [17,34]. Studies of the lumbar spine found that pre-activation of spinal muscles before the onset of movement facilitated better postural stability by increasing muscle spindle sensitivity [35,36].

However, patients’ peak magnitude of supraspinatus were less than control subjects; a finding that is consistent with McMahon and colleague [37] results also among patients with ASI. The early activation of the infraspinatus, and at six times higher peak magnitude compared to control, would have generated the greatest concavity compression force during arm elevation [10,38]. However, the early activation of infraspinatus and supraspinatus delayed the onset of upper trapezius until approximately 45% after movement start. Cumulatively, the early actions of supraspinatus, infraspinatus and delayed onset of trapezius indicated that patients with ASI placed a higher priority on glenohumeral stability before commencing arm mobility.

### Stability at all cost strategy

This study found all seven muscles of patients with ASI peaked primarily at the mid-range of arm elevation. This action reflects the importance they place on establishing glenohumeral stability during the mid-range of arm elevation. However, control subjects spread the peak activities of all seven muscles throughout range, reflecting their ability to optimize recruitment to selective shoulder muscles to generate sufficient concavity compression in mid-range of arm elevation for stability. Our results concur with similar findings among patients with multi-directional shoulder instability and generalized shoulder laxity whom recruited all the shoulder muscles in the mid-range of arm elevation [8]. However, the “stability at all cost” strategy was also observed among asymptomatic individual when they carry a heavy object during arm elevation, demonstrating the versatility of neuro-motor control system [39].

Glousman and coworkers [11] also found that biceps and supraspinatus acted out of phase and with increased activity to compensate for anterior shoulder laxity. Our results confirm their findings. Furthermore, we found a strong peak muscle alliances between infraspinatus and teres major, and supraspinatus and teres major, hinting that other scapula-thoracic muscles may play a significant role as dynamic shoulder stabilizers. We are probably the first to observe that teres major was capable of acting as an additional dynamic stabilizer for patients with ASI. Typically, teres major is an adductor and internal rotation of the shoulder and can contribute to glenohumeral stability at about 90 degrees of elevation. Furthermore, surgical transfer of the teres major after a massive rotator cuff tear found it can pull the humeral head inferiorly and exert antagonistic upward flexion forces to assist with elevation [40]. Among healthy subjects, a strong alliance between teres major with latissimus dorsi, supraspinatus and subscapularis has been reported to also contribute to glenohumeral stability [41]. This finding of the additional mechanical properties of teres major requires further investigation.

Latash & Anson [9] described ‘normal’ neuro-motor patterns are misnomers and said “Central nervous system ‘knows’ how to develop and control movement with respect to unconstrained multi-joint movements…” [pg 59]. There are probably a range of strategies to regulate shoulder joint stability during performance of functional tasks. The central nervous system also favors pre-program neuro-motor strategies to reduce motor redundancy, and for easy and quick action [9]. It has been reported that signals that fire together wire together to generate new sensory neuro-motor maps [42] that may compromise stability in the overhead and apprehension positions. Repetitive practice of altered muscle activations and recruitment of task dependent synergies after ASI may develop to permanent pre-program “stability at all cost” strategy that may remain even after glenohumeral surgical correction. Failure to rectify atypical neuro-motor patterns after shoulder surgery would overload other shoulder muscles, facilitates muscle imbalances and encourage dysfunctional translation of the humeral head on the glenoid, leading to earlier onset of fatigue, movement inefficiency, secondary complications such as scapulathoracic dyskinesis [43-45] and may be a major contribute to recurrent shoulder dislocation [46,47].

One of the limitations of the current study is only seven glenohumeral and scapula-thoracic muscles were studies even though more than 25 muscles are involved in shoulder elevation. Secondly, the set-up of present experimental did not evaluate the apprehension position of the shoulder as such an action could generate abnormal shear forces at the glenohumeral joint that may dislocate the joint. We strongly felt that patients are unlikely to participate in an experimental study that heightens their risk of re-dislocating their unstable shoulder.

ASI is a multi-factorial condition. Our assessments indicated that approximately a third of patients with ASI have positive signs of impingement syndromes with fewer numbers showing presence of biceps tendonitis. Shoulder pain is also a confounding factor that influences the pattern of neuro-motor control. Thus, patients with severe shoulder pain and deficits in external rotation were not included in the present study. Thus, the pattern of neuro-motor control in this study reflects mainly underlying biomechanical factors.

## Conclusions

Muscles are important “sensors of the joint instability” and provide both afferent and efferent signals to the central nervous system to regulate stability of multi-direction joints. This biomechanical study identified atypical neuro-motor control strategies of “stability before mobility” and “stability at all cost” at the glenohumeral joint of patients with ASI as they raised their arm overhead in two planes. The two strategies placed a greater demand on the infraspinatus, recruited the teres major as an additional dynamic stabilizer and shifted the peak activation of external rotator cuff muscles to the mid-range of elevation only. Based on these findings, we recommend that rehabilitation strategies also identify and rectify these abnormal neuro-motor characteristics before the commencement of selective muscle strengthening after shoulder surgical stabilization procedures. Correction of both temporal muscle activation and normalization of their muscle magnitudes of the dynamic shoulder stabilizers may lead to better rehabilitation outcomes and minimize likelihood of shoulder re-dislocation.

## Competing interests

The authors have no competing interests in this article.

## Authors’ contributions

BSR collect the data. All authors read and approved the final manuscript.

## Authors’ information

Suppliers:

a. California Fine Wire Co, P.O. Box 44, Gover Beach, CA 93483–044. USA.

b. Motion Lab, 15045 Old Hammond Hwy, Baton Rouge, LA 7081–1244. USA.

c. Dataq & Windaq Waveform Browser for MMC software, 241 Springside Drive, Alcron, OH44333, USA.

d. Matlab software, TechSource Systems. Pte. Ltd., 10 Ubi Crescent, #0-49, Ubi TechPark, Singapore 40854.

e. SPSS package version 13.0 for Windows http://www-01.ibm.com/software/sg/analytics/spss/

## Pre-publication history

The pre-publication history for this paper can be accessed here:

http://www.biomedcentral.com/2052-1847/5/26/prepub
